# Efficacy of Articaine vs Lignocaine for infiltration anaesthesia during primary molar extractions

**DOI:** 10.12669/pjms.38.4.5343

**Published:** 2022

**Authors:** Song Chen, Jie Xiang, Yan Ji

**Affiliations:** 1 Song Chen, MD, Department of Preventive Dentistry, Nanjing Stomatology Hospital, Medical School of Nanjing University; No. 30, Zhong Yang Road, Nanjing, 210008, Jiangsu Province, P.R. China; 2 Jie Xiang, MD, Department of Preventive Dentistry, Nanjing Stomatology Hospital, Medical School of Nanjing University; No. 30, Zhong Yang Road, Nanjing, 210008, Jiangsu Province, P.R. China; 3 Yan Ji, MD, Department of Preventive Dentistry, Nanjing Stomatology Hospital, Medical School of Nanjing University; No. 30, Zhong Yang Road, Nanjing, 210008, Jiangsu Province, P.R. China

**Keywords:** Articaine, Lignocaine, Extraction, Pain, Anaesthesia, Children, Molar

## Abstract

**Objectives:**

The purpose of this systematic review was to evaluate if articaine has better efficacy as compared to lignocaine when used for infiltration anaesthesia for primary molar extractions.

**Methods:**

The electronic databases of PubMed, Embase, Scopus, BioMed Central, CENTRAL, and Google Scholar were searched up to August 2020. Randomized controlled trials on paediatric patients comparing the infiltration of articaine with lignocaine for extraction of primary molar were included. Pain of extraction and successful palatal/lingual anaesthesia with single buccal infiltration was evaluated.

**Results:**

Six studies were included. We found no difference in pain scores when comparing singular buccal infiltrations of articaine and lignocaine for primary molar extractions. A meta-analysis of extraction pain scores from three studies indicated no statistically significant difference between buccal infiltration of articaine vs combined buccal and palatal/lingual infiltration of lignocaine. Comparing buccal with palatal/lingual infiltration of both articaine and lignocaine with data from three studies, articaine was found to significantly reduce pain scores.

**Conclusion:**

Our review encompassing a limited number of studies suggests that single buccal infiltration of articaine may have a role in primary molar extractions. Articaine may have a better anaesthetic effect compared to lignocaine but the difference may not be clinically relevant.

## INTRODUCTION

Managing paediatric patients for dental procedures needs effective pain control.^1^ Ineffective anaesthesia can instill anxiety and fear in the child and can hamper further treatment.^2^ Since its introduction, lignocaine 2% has been the gold standard anaesthetic agent for both, children and adults. The drug has been the standard against which other anaesthetic agents are compared.^3^

Articaine was first introduced in clinical practice in Germany in 1976.^4^ The drug is unique in that it has a thiophene ring instead of a benzene ring which significantly increases its liposolubility and penetration into tissues. The drug undergoes biotransformation in plasma and liver with further excretion via the kidneys.^5^ It has a short half-life of only 20-40 minutes as compared to about 90 minutes for lignocaine which reduces the risk of systemic toxicity due to multiple injections.^4^,^5^

Primary molar extractions in paediatric patients can evoke significant fear and anxiety. Furthermore, the use of nerve blocks for mandibular molar extractions has disadvantages like prolonged anaesthesia and potential soft tissue injury. In the case of maxillary extractions, palatal injections can be significantly painful owing to the compact mucosa.^6^ Articaine administered via infiltration is known to penetrate the hard and soft tissues more reliably as compared to lignocaine^7^, and thus may be of value in primary molar extractions. Evidence suggests that articaine infiltration produces better anaesthetic success as compared to lignocaine.^8^,^9^ Research on adult patients has also indicated that single buccal infiltration injections of articaine can be used for the extraction of maxillary and mandibular teeth.^7^,^10^ Despite the immense research, evidence on the efficacy of articaine infiltration when used for paediatric dental extractions is very limited. It is unclear if single buccal infiltration of articaine is sufficient for primary molar extraction? and when a similar number of infiltration injections are used, does articaine results in better pain control as compared to lignocaine for primary molar extraction?

Recently, Tong et al^11^ in a review reported no difference in patient-reported pain with articaine and lignocaine when used for paediatric dental procedures. On the other hand, Taneja et al^12^ in a meta-analysis have reported better anaesthetic efficiency with articaine as compared to lignocaine. A drawback of these reviews is that they included trials focussing on the different paediatric dental procedures and using different modes of anaesthesia (infiltration, nerve blocks, or both). To the best of our knowledge, no study has attempted to synthesize evidence on the efficacy of articaine infiltration for primary molar extractions. Therefore, we aimed to conduct a systematic literature search and pool data from studies to evaluate if articaine has better efficacy as compared to lignocaine when used for infiltration anaesthesia for primary molar extractions.

## METHODS

### Search Strategy:

The guideline of the PRISMA statement (Preferred Reporting Items for Systematic Reviews and Meta-analyses)^13^ and the Cochrane Handbook for Systematic Reviews of Intervention^14^ were followed, except for protocol registration. The databases of PubMed, Embase, Scopus, BioMed Central, CENTRAL, and Google scholar were searched by two reviewers from the inception of databases to 15^th^ August 2020. The keywords used were: “articaine”, “lignocaine”, “pediatric”, “children”, “primary molar”, “extraction”, “dental” and “infiltration”. The reviewers screened the search results initially by their titles and abstracts for each database. After identifying potentially pertinent articles, full texts of the articles were extracted and assessed based on the inclusion criteria. Any disagreements were resolved by discussion. The bibliography of included studies were hand searched for any missed references.

### Inclusion criteria:

Only randomized controlled trials (RCTs) were eligible to be included in the review. We further defined the inclusion criteria based on the PICO (Population, Intervention, Comparison, Outcome) framework as follows: *Population*: studies conducted on paediatric patients (<16 years) requiring primary molar extraction. *Intervention*: infiltration anaesthesia with articaine. *Comparison*: infiltration anaesthesia with lignocaine. *Outcomes*: successful anaesthesia and/or extraction pain. Only English language studies were included.

### Exclusion criteria:

- Studies on patients requiring pulpotomy/restorative procedures.
- Studies comparing articaine and lignocaine for inferior alveolar nerve blocks
- Studies comparing infiltration with nerve block
- Studies using conscious sedation and computerized delivery routes.
- Retrospective studies, single-arm studies, and studies not reporting relevant data were also excluded.

Data were extracted by two reviewers independently. Data regarding authors, publication year, study location, study type, age group and gender of the study population, sample size, articaine and lidocaine protocol number of maxillary/mandibular procedures, and study outcomes were extracted.

### Risk of Bias Assessment:

The Cochrane Collaboration risk assessment tool was used for assessing the quality of included RCTs.^14^ The following seven domains were used for quality assessment: random sequence generation, allocation concealment, blinding of participants and personnel, blinding of outcome assessment, incomplete outcome data, selective reporting, and other bias. The study was judged to have a “high”, “unclear”, or “low” risk of bias for each domain. For other bias, the number of operators involved in the study was assessed. Low risk was marked for a single operator while a high risk was marked for ≥2 operators.

### Statistical Analysis:

Studies with similar intervention and control groups were grouped for the analysis, namely, those comparing only buccal infiltration, those comparing combined buccal and palatal/lingual infiltration, and lastly those comparing buccal infiltration of articaine with combined buccal and palatal/lingual infiltration of lidocaine. “Review Manager” (RevMan, version 5.3; Nordic Cochrane Centre [Cochrane Collaboration], Copenhagen, Denmark; 2014) was used for the meta-analysis. Since pain outcomes in the included studies were assessed on different scales, they were pooled using the standardized mean difference (SMD) with 95% confidence intervals (CI). A random-effects model was preferred. Heterogeneity was assessed using the I^2^ statistic. I^2^ values of 25-50% represented low, values of 50-75% medium, and more than 75% represented substantial heterogeneity.

## RESULTS

**Fig.1:**
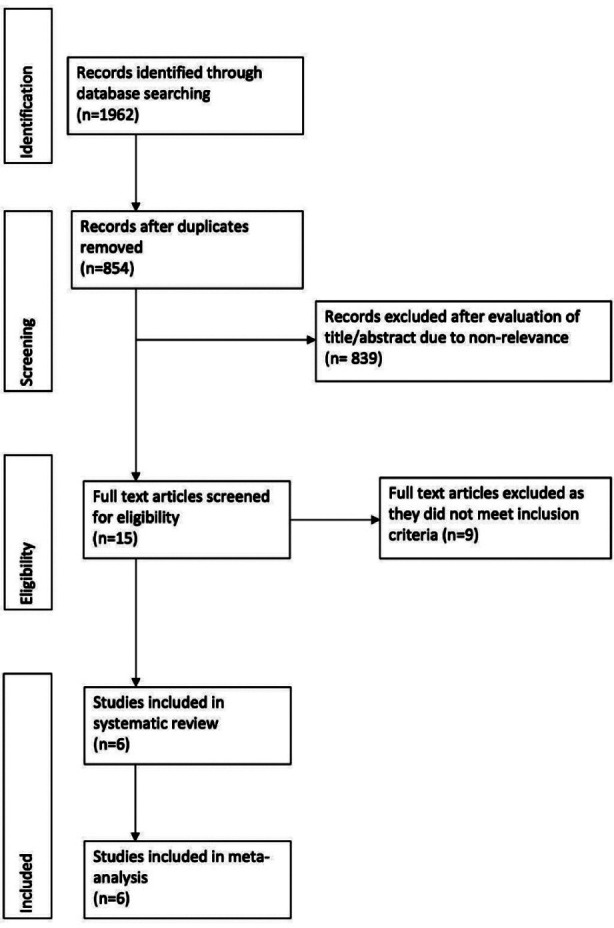
Study flow chart.

The study flow chart is presented in Fig.1. Details of excluded studies^15^-^23^ with reasons are presented in Table-I. A total of 6 RCTs met the inclusion criteria and were included in the analysis^24^-^29^ (Table-II).

**Table-I:** Details of excluded studies.

Study	Reason for exclusion
Arrow et al^15^	Not on primary molar extraction
Badr et al^16^	Not on primary molar extraction
Elheeny et al^17^	Not on primary molar extraction
Sharan et al^18^	Used intra-ligamentary injections
Alzaharani et al^19^	Compared buccal infiltration with nerve block
Afsal et al^20^	Used only nerve blocks
Ramadurai et al^21^	Used only nerve blocks
Malamed et al^22^	Not on primary molar extraction with infiltration anaesthesia
Ram et al^23^	Not on primary molar extraction with infiltration anaesthesia

**Table-II:** Characteristics of included studies.

Author/Year	Country	Age group (y)	Procedure	Study groups	Drug dose	Sample size	Male gender (%)	Maxillary procedures (%)	Pain scale
Massignan et al^24^ / 2020	Brazil	6-10	Primary molar extraction	4% articaine with 1: 100000 epinephrine (BI and LI/PI) 2% lignocaine with 1: 100000 epinephrine(BI and LI/PI)	1.8ml 1.8ml	21 22	45.8 54.2	36.4 63.6	FPS-R
Rathi et al^25^/ 2019	India	7-12	Primary molar extraction	4% articaine with 1: 100000 epinephrine (BI*) 2% lignocaine with 1: 80000 epinephrine (BI*)	1.7ml 1.8ml	50 50	50 50	48 52	WB-FPS
Nair et al^26^/ 2018	India	6-9	Primary molar extraction	4% articaine (BI) 2% lignocaine (BI) 2% lignocaine with 1: 200000 epinephrine (BI and PI)	1.8ml 1.8ml 1.8ml	15 15 15	NR NR NR	100 100 100	VAS
Jaikaria et al^27^/ 2018	India	5-12	Primary molar extraction	4% articaine with 1: 100000 epinephrine (BI*) 2% lignocaine with 1: 100000 epinephrine (BI*)	1.7ml 1.8ml	51 51 51	66.6	100 100	WB-FPS
Kolli et al^28^/ 2017	India	6-14	Primary molar extraction	4% articaine (BI) 2% lignocaine (BI) 2% lignocaine with 1: 80000 epinephrine (BI and PI)	1.7 1.7 1.7	30 30 30	50 53 47	100 100 100	FPS-R
Mittal et al^29^/ 2015	India	5-12	Primary molar extraction	4% articaine with 1: 100000 epinephrine (BI*) 2% lignocaine with 1: 80000 epinephrine (BI*)	1.7ml 1.8ml	52 52	69.2 61.5	100 100	WB-FPS

BI, buccal infiltration; PI, palatal infiltration; LI, lingual infiltration; FPS-R, Facial pain scale-revised; WB-FPS, Wong Baker Facial pain scale; VAS, visual analog scale

* PI/LI given in case of failure to achieve palatal/lingual anesthesia

The age of patients in the trials varied from 5 to 14 years. Four studies^26^-^29^ were conducted only on maxillary primary molar extractions while the remaining^24^,^25^ included both maxillary and mandibular procedures. Two studies^26^,^28^ were three-armed trials wherein only buccal infiltration of articaine and lignocaine was compared with buccal and palatal infiltration of lignocaine. Three studies^25^,^27^,^29^ compared the buccal infiltration of the two drugs but palatal/lingual infiltration was given before extraction, in case of failure to achieve successful palatal/lingual anaesthesia. Lastly, one trial^24^ used both buccal and palatal/lingual infiltration with both articaine and lignocaine groups. There was inter-study variation for the use and dosage of epinephrine.

### Pain of Extraction:

**Fig.2:**
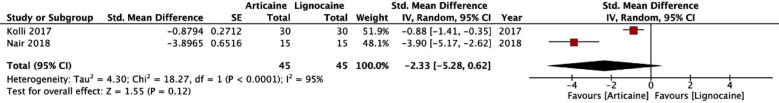
Forest plot of pain scores with buccal infiltration of articaine vs buccal infiltration of lignocaine.

Two studies^26^,^28^ compared singular buccal infiltrations of articaine and lignocaine for primary molar extractions. Both studies were exclusively on maxillary extractions. Pooled analysis indicated no statistically significant difference between articaine and lignocaine for the pain of extraction (SMD: -2.33; 95% CI: -5.28, 0.62; I^2^=95%; p=0.12) (Fig.2).

**Fig.3:**
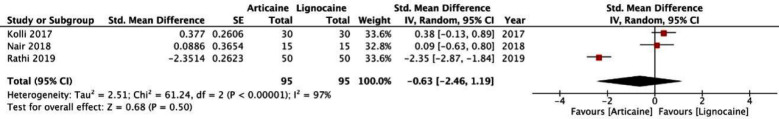
Forest plot of pain scores with buccal infiltration of articaine vs combined buccal and palatal/lingual infiltration of lignocaine.

Three studies^25^,^26^,^28^ compared buccal infiltration of articaine with combined buccal and palatal/lingual infiltration of lignocaine. A meta-analysis of extraction pain scores indicated no statistically significant difference between buccal infiltration of articaine vs combined buccal and palatal/lingual infiltration of lignocaine (SMD: -0.63; 95% CI: -2.46, 1.19; I^2^=97%; p=0.50) (Fig.3).

**Fig.4:**
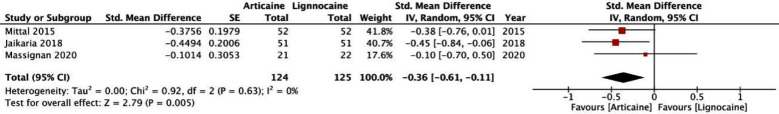
Forest plot of pain scores with combined buccal and palatal/lingual infiltration of articaine vs combined buccal and palatal/lingual infiltration of lignocaine

Buccal and palatal/lingual infiltration of both articaine and lignocaine was compared by three studies.^24^,^27^,^29^ Our meta-analysis indicated statistically significant reduced in pain scores in patients receiving articaine anaesthesia (SMD: -0.36; 95% CI: -0.61, -0.11; I^2^=0%; p=0.005) (Fig.4).

### Successful palatal/lingual anaesthesia:

Three studies^25^,^27^,^29^ reported data on the success of palatal/lingual anaesthesia with sole buccal infiltration of anaesthetic agents. Rathi et al^25^ reported 100% success with articaine but not with lignocaine. All patients in the lignocaine group of their trial were given palatal/lingual infiltration before extraction. On the other hand, Jaikaria et al^27^ and Mittal et al^29^ did not report successful palatal anaesthesia with either drug. Palatal anaesthesia was noted in only one patient in the articaine group of Mittal et al.^29^ All remaining patients in both groups of the two trials required additional palatal infiltration before extraction.

### Risk of bias:

**Fig.5:**
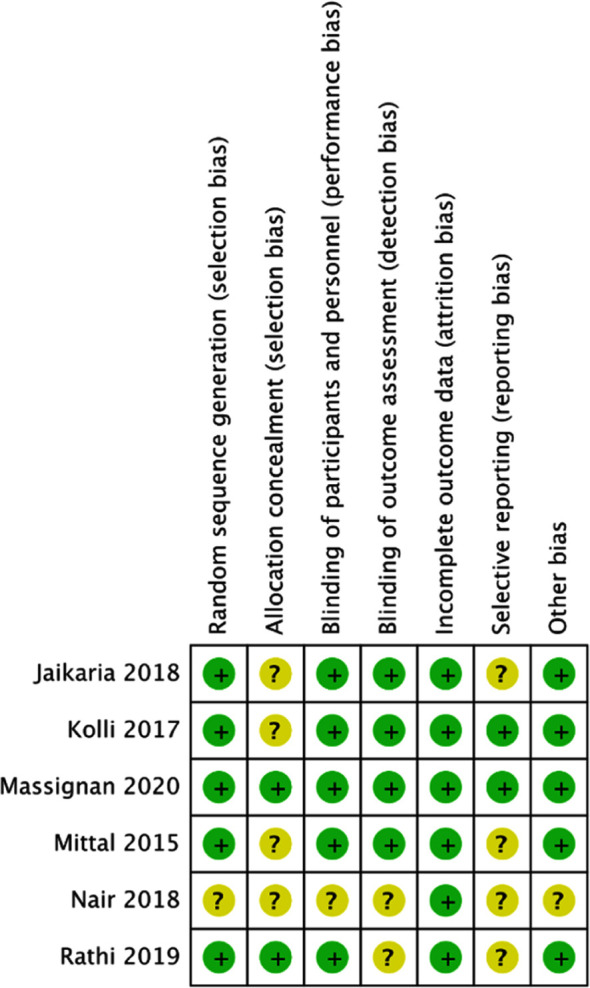
Risk of bias analysis. Yellow circles denote unclear risk of bias, Green circles denote low risk of bias.

The risk of bias summary of the included studies is presented in Fig-5. Allocation concealment was not clearly described in four studies.^26^-^29^ Blinding of both personnel and outcome assessors was not mentioned in the trial of Nair et al.^26^ Only two studies^24^,^28^ were pre-registered to assess reporting bias. The trial of Nair et al^26^ did not specify the number of operators involved in the study.

## DISCUSSION

Despite the common use of local anaesthetics in dental practice, the technique does not produce absolute success.^30^ Out of the two most common anaesthesia techniques, infiltration injections are less painful than nerve blocks in children, except for palatal injections which can elicit significant pain.^31^,^32^ Given the advantages of infiltration anaesthesia and its established efficacy, there is a need for further enhancing its effect by more effective drugs. In this context, it is important to know if articaine results in better anaesthetic efficacy compared to lignocaine.

The high lipid solubility of articaine which increases its hard and soft tissue permeability has encouraged research on single buccal infiltrations of the drug for dental extractions. Uckan et al^33^ in one of the earliest studies demonstrated that maxillary extractions may be possible without palatal injections. In a double-blind RCT, Sandilya et al^34^ have also indicated that single buccal infiltration of articaine is sufficient for the extraction of maxillary premolars in adults. In a recent study, Majid et al^7^ concluded that single buccal infiltration of articaine is no different from buccal and lingual infiltration of the drug and sole buccal infiltration can be used for extraction of mandibular premolars. Singular buccal injections for extractions may have potential advantages in paediatric patients by reducing the number of injections, eliminating painful palatal injections, and thereby improving the comfort of the procedure.

As the trials included in our review differed in the use of buccal and palatal/lingual injections with either drug, we grouped them into similar sub-groups to better elucidate current evidence. In the first meta-analysis, we compared a singular buccal infiltration of articaine and lignocaine for primary maxillary molar extractions. While our results indicated to the difference between the two groups, a closer look at the forest plot indicates that both the trials (Nair et al^26^ and Kolli et al^28^) reported significantly lower pain scores with articaine as compared to lignocaine since the upper end of 95% CI were below 0 for both trials. The pooled SMD was -2.33 with the lower end of the 95% CI at -5.28 and the upper end very close to zero at 0.62. Thus, despite the insignificant results, the values of the 95% CI of our analysis suggest that articaine may have a role in primary molar extractions when used for singular buccal infiltration. This is further supported by the results of the second meta-analysis where we compared single buccal infiltration with combined buccal and palatal/lingual infiltration of lignocaine. Our results demonstrated no statistically significant difference in pain scores with either group. Therefore, single buccal infiltration of articaine may be equally efficacious as combined buccal and palatal/lingual infiltration of lignocaine. However, the results must be interpreted with caution as only a limited number of studies were available for analysis. Evidence is further obscured by the conflicting results of successful palatal/lingual anaesthesia in the included studies. Only three trials evaluated this outcome with one reporting 100% success with articaine and two demonstrating no effect of the drug. Such contradictory results are difficult to explain given the fact all three trials used similar concentrations of articaine. The strength of evidence on single buccal injections of articaine for maxillary extractions in adults has also been questioned. Cui et al^35^ in a meta-analysis on adult patients have concluded that despite individual studies reporting no increase in pain scores for extraction of maxillary teeth with single buccal infiltration of articaine, the success of maxillary extractions is reduced when palatal injections are omitted. Even in our review, we are unable to conclude on the efficacy of single buccal infiltration of articaine.

It is known that the higher the degree of binding of the local anaesthetic molecule with the nerve membrane, the more prolonged is the anaesthetic effect along with better pain control.^5^ Thus, even with the same injection technique, articaine may theoretically result in better anaesthetic effect as compared to lignocaine.^9^ Such results have been confirmed by Narendrababu et al^36^ wherein articaine was found to be more efficacious than lignocaine for anaesthesia of teeth with irreversible pulpitis. To evaluate such outcomes in paediatric patients, in the third sub-group of our review, we compared the anaesthetic efficacy of articaine and lignocaine with a similar number of infiltration injections. Our analysis indicated that articaine infiltrated buccally and palatally/lingually significantly reduces pain scores as compared to similar injections of lignocaine, albeit with a very small effect size. Similar results have been noted by Taneja et al^12^ for paediatric dental procedures. The authors reported small reduction in pain scores on the facial pain scale (FPS) and the Visual Analog Scale (VAS) with articaine as compared to lignocaine. The clinical relevance of such a small difference is questionable, especially in paediatric patients.

### Limitations of this review:

Firstly, only a limited number of studies were available for inclusion in our review. The analysis may not have been adequately powered to detect significant differences. Secondly, there was variation in the pain scales used by the included trials. This was, however, compensated with the use of SMD to calculate the summary effect. Secondly, pain scores in children can be very subjective.^37^ Pain in children can be influenced by several factors like age, gender, anxiety, current dental symptoms, and past experience.^30^ The presence of these unaccounted confounding factors could have skewed the results. Thirdly, there was inter-study heterogeneity in the included trials for the included age groups, dosage of the drugs, and tooth to be extracted. The resorption stage of the primary molar was varied and the length of the remaining root could have influenced the complexity of the extractions. Lastly, important variables like pain on injection, the onset of anaesthesia, success rates of palatal/lingual anaesthesia, and adverse events were not universally reported in the included studies. This restricted the ability of this review to provide an in-depth comparison of the two drugs.

## CONCLUSION

Our review encompassing a limited number of studies suggests that single buccal infiltration of articaine may have a role in primary molar extractions. Also, articaine may have a better anaesthetic effect compared to lignocaine but the difference may not be clinically relevant. Current evidence is weak and there is a need for further high-quality RCTs with a large sample size.

### Authors’ contributions:

**SC:** Conceived and designed the study.

**JX & YJ:** Collected the data and performed the analysis.

**SC:** Was involved in the Writing of the manuscript and is responsible for integrity of the study.

**YJ:** Edited the manuscript.

All authors have read and approved the final manuscript.

## References

[1] Jorgenson K, Burbridge L, Cole B (2020). Comparison of the efficacy of a standard inferior alveolar nerve block versus articaine infiltration for invasive dental treatment in permanent mandibular molars in children:a pilot study. Eur Arch Paediatr Dent.

[2] Kuscu OO, Akyuz S (2008). Is it the injection device or the anxiety experienced that causes pain during dental local anaesthesia?. Int J Paediatr Dent.

[3] Lagan G, McLure HA (2004). Review of local anaesthetic agents. Curr Anaesth Crit Care.

[4] Snoeck M (2013). Articaine:A review of its use for localand regional anesthesia. Local Reg Anesth.

[5] Hopman AJG, Baart JA, Brand HS (2017). Articaine and neurotoxicity-A Review. Br Dent J.

[6] Hassan S, Sripathi Rao B, Sequeria J, Rai G (2011). Efficacy of 4% articaine hydrochloride and 2% lignocaine hydrochloride in the extraction of maxillary premolars for orthodontic reasons. Ann Maxillofac Surg.

[7] Majid OW, Muhammad ZA (2019). Effectiveness of Articaine Buccal Infiltration Anesthesia for Mandibular Premolar Extraction:A Randomized, Double-Blind, Placebo-Controlled Clinical Trial. J Oral Maxillofac Surg.

[8] Soysa NS, Soysa IB, Alles N (2019). Efficacy of articaine vs lignocaine in maxillary and mandibular infiltration and block anesthesia in the dental treatments of adults:A systematic review and meta-analysis. J Investig Clin Dent.

[9] Zhang A, Tang H, Liu S, Ma C, Ma S, Zhao H (2019). Anesthetic Efficiency of Articaine Versus Lidocaine in the Extraction of Lower Third Molars:A Meta-Analysis and Systematic Review. J Oral Maxillofac Surg.

[10] Bataineh AB, Nusair YM, Al-Rahahleh RQ (2019). Comparative study of articaine and lidocaine without palatal injection for maxillary teeth extraction. Clin Oral Investig.

[11] Tong HJ, Alzahrani FS, Sim YF, Tahmassebi JF, Duggal M (2018). Anaesthetic efficacy of articaine versus lidocaine in children's dentistry:a systematic review and meta-analysis. Int J Paediatr Dent.

[12] Taneja S, Singh A, Jain A (2020). Anesthetic Effectiveness of Articaine and Lidocaine in Pediatric Patients During Dental Procedures:A Systematic Review and Meta-Analysis. Pediatr Dent.

[13] Moher D, Liberati A, Tetzlaff J, Altman DG, PRISMA Group (2009). Preferred Reporting Items for Systematic Reviews and Meta-Analyses:The PRISMA Statement. PLoS Med.

[14] Higgins J, Thomas J, Chandler J (2019). Cochrane Handbook for Systematic Reviews of Interventions. Version 6. Cochrane.

[15] Arrow P (2012). A comparison of articaine 4% and lignocaine 2% in block and infiltration analgesia in children. Aust Dent J.

[16] Badr S, Elsharkawy R, Elsholkamy M (2013). The effectiveness of articaine versus lidocaine as infiltration anesthesia for mandibular posterior teeth in pediatric patients. Egypt Dent J.

[17] Elheeny AAH (2020). Articaine efficacy and safety in young children below the age of four years:An equivalent parallel randomized control trial. Int J Paediatr Dent.

[18] Sharan S, Goswami M, Kaul R, Rahman B, Farooq S (2018). Comparative evaluation of effectiveness of intraligamentary injection technique using articaine and lidocaine for extraction of primary mandibular posterior teeth. Int J Pedod Rehabil.

[19] Alzahrani F, Duggal MS, Munyombwe T, Tahmassebi JF (2018). Anaesthetic efficacy of 4% articaine and 2% lidocaine for extraction and pulpotomy of mandibular primary molars:an equivalence parallel prospective randomized controlled trial. Int J Paediatr Dent.

[20] Afsal MM, Khatri A, Kalra N, Tyagi R, Khandelwal D (2019). Pain perception and efficacy of local analgesia using 2% lignocaine, buffered lignocaine, and 4% articaine in pediatric dental procedures. J Dent Anesth Pain Med.

[21] Ramadurai N, Gurunathan D, Samuel AV, Subramanian E, Rodrigues SJL (2019). Effectiveness of 2% Articaine as an anesthetic agent in children:randomized controlled trial. Clin Oral Investig.

[22] Malamed SF, Gagnon S, Leblanc D (2000). A comparison between Articaine HCl and Lidocaine HCl in pediatric dental patients. Pediatr Dent.

[23] Ram D, Amir E (2006). Comparison of articaine 4% and lidocaine 2% in paediatric dental patients. Int J Paediatr Dent.

[24] Massignan C, Silveira Santos P, Cardoso M, Bolan M (2020). Efficacy and adverse events of 4% articaine compared with 2% lidocaine on primary molar extraction:A randomised controlled trial. J Oral Rehabil.

[25] Rathi N V., Khatri AA, Agrawal AG, Sudhindra Baliga M, Thosar NR, Deolia SG (2019). Anesthetic efficacy of buccal infiltration articaine versus lidocaine for extraction of primary molar teeth. Anesth Prog.

[26] Nair M, Jeevanandan G, Mohan M (2018). Comparing the efficiency of 2% lidocaine and 4% articaine as a local anesthetic agent in children. Asian J Pharm Clin Res.

[27] Jaikaria A, Thakur S, Singhal P, Chauhan D, Jayam C (2017). Comparative evaluation of the efficacy of 4% articaine and 2% lidocaine in children during the primary maxillary molar extractions. Indian J Oral Heal Res.

[28] Kolli NR, Nirmala SVSG, Nuvvula S (2017). The effectiveness of articaine and lidocaine single buccal infiltration versus conventional buccal and palatal injection using lidocaine during primary maxillary molar extraction:A randomized control trial. Anesth Essays Res.

[29] Mittal M, Sharma S, Kumar A, Chopra R, Srivastava D (2015). Comparison of Anesthetic Efficacy of Articaine and Lidocaine During Primary Maxillary Molar Extractions in Children. Pediatr Dent.

[30] Nakai Y, Milgrom P, Mancl L, Coldwell SE, Domoto PK, Ramsay DS (2000). Effectiveness of local anesthesia in pediatric dental practice. J Am Dent Assoc.

[31] Jones CM, Heidmann J, Gerrish AC (1995). Children's ratings of dental injection and treatment pain, and the influence of the time taken to administer the injection. Int J Paediatr Dent.

[32] Tudeshchoie DG, Rozbahany NA, Hajiahmadi M, Jabarifar E (2013). Comparison of the efficacy of two anesthetic techniques of mandibular primary first molar:A randomized clinical trial. Dent Res J (Isfahan).

[33] Uckan S, Dayangac E, Araz K (2006). Is permanent maxillary tooth removal without palatal injection possible?. Oral Surgery, Oral Med Oral Pathol Oral Radiol Endodontol.

[34] Sandilya V, Andrade N, Mathai P, Aggarwal N, Sahu V, Nerurkar S (2019). A randomized control trial comparing buccal infiltration of 4% articaine with buccal and palatal infiltration of 2% lignocaine for the extraction of maxillary premolar teeth. Contemp Clin Dent.

[35] Cui L, Zhang Z, Huang J, Yin D, Xu L (2018). Extraction of permanent maxillary teeth without palatal injection:a meta-analysis. Oral Surg Oral Med Oral Pathol Oral Radiol.

[36] Nagendrababu V, Duncan HF, Whitworth J (2020). Is articaine more effective than lidocaine in patients with irreversible pulpitis?An umbrella review. Int Endod J.

[37] Stahlschmidt L, Hubner-Mohler B, Dogan M, Wager J (2019). Pain Self-Efficacy Measures for Children and Adolescents:A Systematic Review. J Pediatr Psychol.

